# Artificial intelligence-based positive youth development intervention protocol in school settings

**DOI:** 10.3389/fpsyg.2026.1901161

**Published:** 2026-08-04

**Authors:** Mehmet Akif Karaman, Ashraf Mahmud

**Affiliations:** Department of Liberal Arts, American University of the Middle East, Egaila, Kuwait

**Keywords:** AI-supported intervention, feasibility design, positive youth development, school belonging, self-regulation

## Abstract

Positive Youth Development (PYD) conceptualizes youth as agentic, developmentally flexible individuals whose functioning reflects dynamic person–context transactions. Rapid developments in AI now offer new opportunities to support counselors and extend intervention-based programs. The purpose of this paper is to outline an eight-week AI-supported PYD intervention for high school students and frame a feasibility-oriented mixed-method outcome research design. The model integrates daily AI micro-nudges, short voluntary micro-coaching dialogues, and one weekly 10–15-min counselor mini-session. Trait-level outcomes (self-regulation and school belonging) are assessed pre–post; state-level micro-regulation is assessed daily via Ecological Momentary Assessment (EMA). Quantitative analysis uses Lakens’s generalized within-subject effect-size formulation and baseline trend-corrected Tau-U; qualitative analysis uses deductive thematic analysis. The design is intended to demonstrate whether mechanism-consistent developmental micro-arrangements occur in naturalistic conditions rather than to test causal efficacy. This paper provides a research-ready protocol for future randomized controlled trials in AI-supported school-based intervention program.

## Introduction

Positive Youth Development (PYD) has emerged as one of the most empirically consolidated meta-frameworks in contemporary developmental research. Unlike pathology-oriented approaches that conceptualize youths primarily as carriers of vulnerability or risk, PYD advances a strength-based, asset-focused approach grounded in developmental systems thinking (Benson et al., 2006; Lerner and Callina, 2015; Waters, 2011). This framework conceptualizes adolescents as proactive and adaptable agents embedded within a complex web of intersecting systems, notably families, schools, peers, communities, and increasingly, digital environments (Fitzpatrick et al., 2017; Lerner and Callina, 2015; Potts et al., 2025). This perspective essentially transforms the central objective of youth intervention research: rather than focusing primarily on mitigating negative behaviors, the scientific target shifts toward the active cultivation of developmentally significant assets, such as competence, confidence, social connectedness, and self-regulation (de Ridder et al., 2012). These outcomes represent proactive markers of thriving rather than mere absences of pathology, necessitating intervention designs capable of detecting developmental growth rather than just clinical amelioration (Abdul Kadir and Rusyda, 2022).

While PYD offers a robust theoretical framework, its practical implementation in schools is rarely hindered by the counselor’s expertise, but rather frequently bottlenecked by structural constraints: large caseloads, fragmented schedules, limited institutional bandwidth, and the need for sustained bi-directional relational engagement (Girio-Herrera et al., 2019; Hilts et al., 2019). Despite strong theoretical consensus, a severe translational bottleneck persists in the field. Traditional PYD interventions such as manualized SEL curricula, after-school programs, and weekly counseling groups are effective but are resource-intensive, count heavily on retroactive self-reporting, and struggle with ecological validity (i.e., skills gained in a controlled classroom often fail to transfer to the unpredictable environments of the school hallways or the digital sphere). Recent studies also suggest that the effective implementation of PYD interventions frequently requires deliberate cultural tailoring (Harris and Cheney, 2015), gender-sensitive framing (Gomez-Baya et al., 2023), and multi-layered evaluation models (Eichas et al., 2010), conditions that many school counseling units struggle to operationalize on a continuous basis.

To overcome these constraints, emerging studies are increasingly turning toward digital interventions. Large-scale international trials like the European SEL4@ll program are investigating the efficacy of serious games and interactive platforms to promote and enhance social–emotional competencies outside the classroom. Methodologies like Just-In-Time Adaptive Interventions (JITAIs) and Ecological Momentary Assessment (EMA) are gaining traction (Nahum-Shani et al., 2018). These emerging approaches recognize that traditional interventions like scheduled weekly session often struggle to offer real-time responses and support, critically when a youth is experiencing a pertinent stressor or social opportunity. Recent growth in artificial intelligence (AI) and machine learning (ML) approaches in psychology and neuroscience now offers promising new possibilities for more targeted and personalized intervention in data-driven behavioral care (Benrimoh et al., 2025). AI represents the critical bridge to operationalizing these advanced, ecologically valid PYD models at scale. While early, rigid digital health tools (e.g., static applications or modules) suffered from high attrition, conversational AI scales up the automated delivery of high-frequency, low-barrier developmental affordances. Standard “one-size-fits-all” SEL curricula are not personalized to an adolescent’s specific needs and contexts, which limits adolescent engagement. By analyzing EMA data, interaction timing, and micro-choices, AI can dynamically tailor its scaffolding. For example, if an adolescent consistently reports low belonging on Tuesday, the AI can deliver customized social-connection nudges on Mondays, creating a highly personalized developmental trajectory. Adolescents experience momentary dysregulation and rapid mood shifts from peer conflict, bullying, or digital anxiety (e.g., fear of missing out). In these acute moments, AI provides immediate micro-coaching and reflective containment, guiding youth through a self-regulatory process before a situation deteriorates. Within the school setting, AI is not intended as a replacement for the counselor relationship, but as a dosage amplifier. AI can manage the high-frequency micro-interaction burden, routine tasks, and administrative oversight, thus, freeing counselors to preserve relational depth with students while significantly expanding developmental opportunities across the broader youth population.

Recent advances (e.g., Fan et al., 2025; Su et al., 2024; Zhu et al., 2024) in AI-assisted delivery systems offer novel design affordances that can alleviate counselor burden while preserving the relational core of school counseling. Micro-interaction models, such as brief reflective prompts, self-regulation nudges, or short cognitive-scaffolding dialogues, can be delivered autonomously. This may support the high-frequency dosage intended to promote developmental change without exhausting human resources (Zhu et al., 2024). The utility of AI in this context does not rely on its ability to perform deep clinical interpretation or understand adolescents accurately; rather, its value lies in delivering frequent, properly framed micro-affordances that youth can convert into meaningful developmental practice. For PYD, this matters profoundly, because PYD outcomes such as self-regulation and social connectedness are not passively absorbed classroom-style content but enacted practices requiring fresh-in-context repetition. Ultimately, counselors define the developmental arc, while AI executes the micro-dosage between relational touchpoints.

This paper addresses this methodological gap by defining an intervention design scaffold rather than reporting on clinical efficacy. Specifically, we describe an eight-week, school-embedded PYD intervention for high school students (aged 14–18) that integrates a dual-channel AI augmentation architecture: (a) daily motivational interviewing (MI)-based micro-nudges aligned with weekly developmental objectives, and (b) short, reflective micro-coaching dialogues triggered voluntarily by user or when engagement lapses. This AI architecture is anchored by a weekly synchronous 10–15-min counselor mini-session to maintain relational containment and fidelity supervision. By presenting a feasibility-oriented research blueprint rather than a commercial or finalized clinical product, this paper contributes a rigorous, research-ready protocol backbone that future randomized controlled trials can directly adapt, parameterize, and empirically test.

### Theoretical grounding and the current state of PYD

The PYD is grounded in relational developmental systems theory, which conceptualizes youth as active, flexible, and contextually embedded agents whose developmental growth emerge through dynamic, individual–environment transactions (Lerner et al., 2005). Many brain areas, particularly the prefrontal cortex, which controls complex cognition and executive functions, follow a protracted maturation period until late adolescence (Kolk and Rakic, 2022; Mahmud and Karaman, 2026). While this prolonged plasticity period increases vulnerability to environmental adversities, it also creates a developmental window during which youths are responsive to positive environmental scaffolding and capacity-building interventions. PYD rejects the historical assumption that adolescence is primarily a period of vulnerability, impulsivity, or deficit. Instead, it frames this developmental window as a period of enhanced opportunities and adaptive plasticity, where supportive ecological conditions can promote thriving rather than merely prevent dysfunction (Lerner, 2009). These conceptual foundations make PYD exceptionally well-suited for rigorous empirical outcome research, as developmental targets and outcomes are directly observable, modifiable, and measurable, rather than latent, moralistic, or aspirational.

PYD models are typically articulated in terms of multi-dimensional “assets,” such as Lerner’s “5 Cs” (Competence, Confidence, Connection, Character, Caring) (Roth and Brooks-Gunn, 2003). In recent decades, the field of PYD has shifted away from deficit-based models, which focused on mitigating adolescent risk behaviors like substance use, delinquency, and school dropout, toward strength-based, ecological frameworks. Recent theoretical advancements have expanded Lerner’s seminal “5 Cs” to include a 6th C (Contribution) and, increasingly, a 7th C (Creativity or Critical Consciousness) to address adaptive problem-solving within increasingly complex social contexts (Lerner et al., 2021; Shirzad et al., 2026). Developmental researchers now emphasize that constructs like purpose, hope, and social–emotional learning (SEL) are dynamic capacities rather than static traits, requiring continuous, real-world practice (Bowers and Bowers, 2023). Consequently, a successful transition to adulthood requires the episodic education and the continuous integration of self-regulation and social competence into the adolescent’s daily life. However, not all PYD indicators are equally sensitive to short-term intervention exposure in school settings. Broad outcomes such as “character” or “identity integration” typically emerge longitudinally (Negru-Subtirica et al., 2017), whereas regulatory processes (e.g., attention, emotion modulation, behavioral self-management) can shift proximally and repeatedly across daily school interactions (Waters, 2011; Zhang et al., 2015). For feasibility-oriented outcome designs, these regulatory constructs offer two distinct advantages: (a) they can be efficiently indexed using short Ecological Momentary Assessment (EMA) indicators, and (b) they fluctuate in real time within authentic environments. Thus, this protocol treats self-regulation not merely as an abstract trait, but as an actionable discrete set of micro-skills that the youth can practice, rehearse, and strengthen through repeated AI-driven affordances.

The second targeted PYD domain in this protocol, social connectedness, is similarly grounded in relational systems theory. During adolescence, peer significance, relational belonging, and perceived social support emerge as powerful predictors of both psychological well-being and academic engagement (Wentzel, 2017). Social connectedness is framed not as superficial “popularity” or “likability,” but as the subjective perception of being embedded in meaningful relational networks. This perception can be actively cultivated through discrete interactional shifts, such as attuning to social cues, performing micro-prosocial behaviors, or accurately decoding and interpreting peers’ emotional signals, which could be target for AI-scaffolded micro-interventions. Consequently, social connectedness serves as a synergistic secondary PYD outcome domain that is theoretically linked to regulatory practice: effectively self-regulated youth tend to interact more adaptively (Moschko et al., 2023), and these adaptive interactions, in turn, reinforce social belonging (Schlegel, 2015).

The integration of these two domains, self-regulation and social connectedness, creates a theoretically coherent, methodologically rigorous, and ecologically viable pairing for school-based implementation. Both domains have clearly defined behavioral manifestations, both exhibit sensitivity to short-term proximal dosage, and both are developmentally central for adolescents aged 14–18. Within the context of a feasibility-oriented outcome research, this dual-domain framework provides a tractable analytic target that seamlessly bridges abstract PYD theory with operationally measurable and intervention-sensitive outcomes.

By mapping specific programmatic features to Lerner’s 5 Cs, the AI is constrained and directed by established developmental theory (Bowers and Bowers, 2023; Lerner et al., 2005). It does not operate as an open-ended conversational agent, but as a highly targeted developmental scaffold (Fan et al., 2025). Each micro-nudge and coaching dialogue is mathematically weighted to prompt behaviors that actively build Competence, Confidence, Connection, Character, or Caring. Consequently, the technology serves as a precision instrument to operationalize the exact individual-environment transactions that relational developmental systems theory identifies as the engine of youth thriving (Lerner and Callina, 2015; Su et al., 2024).

### Model definition

The model is conceptualized as a developmental delivery architecture wherein human counselors establish the relational frame and developmental arc, while AI system executes the high-frequency micro-dosage required for proximal skill enactment. Critically, this intervention does not conceptualize AI as a counselor analog, therapist substitute, or diagnostic component, but as a systemic dosage amplifier. Counselors maintain primary responsibility for relational containment, while the AI autonomously delivers the high-volume, micro–developmental practice opportunities that human counselors cannot feasibly scale. This approach is grounded in the developmental reality that self-regulation and social connectedness are not cultivated through episodic learning, but through continuous, real-world repetition. The architecture therefore mirrors the mechanisms of motor-skill acquisition: micro-practice → consolidation → transfer. This counselor–AI co-delivery framework makes this repetition feasible within standard school constraints.

The intervention is deployed throughout eight consecutive school weeks. Each week focuses on one thematic developmental objective (see Table 1) and offers a synchronous 10–15-min counselor mini-session. This mini session serves as a vital “anchor point,” grounding the week’s developmental target in a supportive relational context while allowing the counselor to check ecological fit, safety, and progress. The remainder of the weekly dosage is delivered digitally through daily MI-style micro-nudges (e.g., ≤ 40 words). The sequence is intentionally graded to build capacity over time: initial weeks emphasize foundational awareness and micro-choice recognition; intermediate weeks prioritize active skill application in real-life contexts; final weeks focus on value mapping, identity congruence, and future transfer. In essence, the intervention is paced not by superficial “topic rotation,” but through the cumulative scaffolding of practice complexity.

**Table 1:** Structured eight-week progression of developmental objectives and measurable indicators.

Week	Theme	Weekly skill focus	Measurable outcome wording	Measurement schedule
0				Weeks −2 to −1: Consent, demographics, BSCS/SSBS (pre), two-week daily EMA baseline (Phase A)
1	Awareness	Noticing internal states & situational triggers	Student(s) can label at least one emotional state + one situational trigger per day	
2	Micro-Choices	Identifying ‘choice points’ in daily routines	Student(s) can describe one moment/day where a micro-choice was made intentionally	
3	Regulation Moves	Using micro-regulation strategies in real time	Student(s) can report using at least one regulation move during a difficult moment	
4	Social Signals	Reading emotional/social cues in peers	Student(s) can identify one social cue observed in a peer interaction per day	Weeks 1–8: Daily EMA, weekly mini-session, fidelity log
5	Connection Behaviors	Performing pro-social action steps	Student(s) can report one specific pro-social action per day	
6	Real-World Practice	Applying skills in naturalistic school contexts	Student(s) can name one applied skill used in a school scenario that occurred	
7	Integration & Identity	Linking skills to values and identity	Student(s) can articulate one personal value and how a behavior reflected it	Week 8: BSCS/SSBS (post)
8	Transfer & Future Self	Projecting skills into future-oriented plans	Student(s) can describe one future scenario where learned skills will be used	

The AI augmentation is delivered through two channels. Channel 1 deploys daily micro-nudges aligned with the overarching weekly objective; these function as single-turn reflective invitations that increase the density of developmental prompts across the student’s daily ecological environment. Channel 2 consists of short-form (3–5 turn) micro-coaching dialogues, triggered either by voluntarily user initiation or automatically triggered following a 24-h lapse in engagement. Both channels are strictly reflective, offering no directive advice, clinical instruction, or evaluative feedback. The goal is to systematically increase the frequency with which the student monitors internal states, detects micro-choice moments, decodes social signals, and reflects on prosocial behaviors. Over the 8 weeks, this dual-channel architecture aims to expose the student to dozens of micro self-regulation enactments and relational affordances that incrementally accumulate to drive sustained and measurable developmental change.

In this intervention, the counselor supervises fidelity, not content generation. The synchronous weekly mini session(s) serves to anchor and facilitate relational connection, provide developmental interpretation, and maintain clinical boundaries. To support this, counselors receive an auto-generated weekly summary of the student’s (a) micro-nudge delivery and engagement, (b) micro-coaching activity, and (c) reflective note submissions. The counselor’s task is not to manually “grade” or “correct” but to contextualize the AI-facilitated micro-work within the student’s authentic relational ecology, and to intervene if emotional distress or dysregulation emerges. Ultimately, this design upholds the core ethical framework that the AI augments the intervention dosage, but human counselors remain fundamental anchors of the developmental and relational process.

## Method

### Setting and participants

The intervention is designed for implementationwithin mainstream high school counseling settings serving adolescents aged 14–18. This ecological frame is intentional. As a feasibility study, the sample size is determined by the need to evaluate implementation applicability and estimation precision rather than statistical power for clinical efficacy. A target sample of 40–60 students from routine school caseloads, is sufficient to yield narrow confidence intervals (CIs) for recruitment/retention/compliance and to visualize heterogeneous individual-level micro-trajectories within an authentic delivery context. Feasibility will be evaluated against four pre-specified operational thresholds: (a) a recruitment rate ≥ 70% of eligible students; (b) a retention rate of ≥ 80% (defined as completing both pre- and post-intervention trait measures); (c) a median EMA compliance of ≥ 60% for daily prompts (1–7 Likert scale) across Weeks 1–8; (d) an intervention fidelity of ≥ 75% (defined as weeks where all three checklist items are met). These thresholds were derived from established benchmarks in the feasibility and pilot trial literature (Billingham et al., 2013; Eldridge et al., 2016). The 70% recruitment and 80% retention rates are widely adopted as minimum indicators of a viable study pipeline for a subsequent full-scale trial. The 60% EMA compliance threshold reflects the empirically observed lower bound of acceptable ecological momentary data completeness in adolescent digital health studies (Shiffman et al., 2008); compliance below this level produces phase lengths insufficient for reliable Tau-U estimation. The 75% fidelity threshold is grounded in the intervention science literature’s recommendation that at least three-quarters of protocol sessions be delivered as specified before efficacy conclusions can be considered (Bellg et al., 2004). Together, these benchmarks ensure that a positive feasibility verdict is substantively meaningful rather than nominally so.

Participation is voluntary, contingent upon the student’s willingness to engage in daily reflective micro-interactions for 8 weeks. Inclusion criteria are intentionally minimal to maximize ecological validity: must (a) possess sufficient functional literacy to interpret daily reflective prompts, (b) have access to a digital interface (e.g., mobile device), and (c) be available for one weekly 10–15 min synchronous mini-session with a counselor. Exclusion criteria are guided by safety and feasibility logic rather than clinical diagnostics. Students presenting with acute crisis indicators (e.g., active suicidal intent, imminent risk of harm, or major protective disclosure) are not enrolled into this model because such circumstances require immediate professional care with crisis protocols. The purpose of this design is not to triage or treat acute dysregulation but to examine whether an AI-supported PYD scaffold can be realistically implemented within an ordinary school setting.

To ensure participant safety, any Adverse Events (AEs) including disclosures of harm, acute distress, or technology misuse will be monitored, documented, and reported to the responsible researcher within 24 h. AEs trigger immediate counselor escalation in accordance with institutional safeguarding policies; serious AEs are logged and reviewed by the school’s safeguarding lead. The AI system is structurally restricted from issuing behavioral directives; all guidance remains purely reflective and strictly under human counselor’s clinical oversight.

### Measures

Trait-level self-regulation will be assessed using the Brief Self-Control Scale (BSCS; Tangney et al., 2004), a short 13-item self-report instrument, psychometrically validated across 11 languages, and widely used as a robust proxy for dispositional self-regulatory capacity in adolescent and adult populations. Items are scored on a 5-point Likert scale ranging from 1 (*not at all like me*) to 5 (*very much like me*). The BSCS captures stable individual differences in the ability to modulate attention, emotion, impulses, and behavior, making it appropriate for detecting trait-level shifts in response to developmental scaffolding. A sample item is, “I wish I had more self-discipline.” The BSCS has demonstrated good internal consistency, (*α* = 0.83; Tangney et al., 2004). In a feasibility design, the objective is not to optimize latent factor modeling, but to index whether directional improvement is present at the level of standardized mean difference. Consequently, the BSCS will be administered one week prior to intervention onset and immediately post-intervention.

Trait-level social connectedness will be indexed using the Simple School Belonging Scale (SSBS; Whiting et al., 2017), a 10-item instrument that quantifies the subjective perception of being psychologically included, relationally valued, and socially embedded within the school’s peer ecology. A sample item is, “I feel like my ideas count at [school name].” School belonging is one of the most tightly coupled environmental predictors of both academic persistence and socioemotional well-being in adolescence. The SSBS functions as a proximal phenotype of this social connection, and changes in SSBS scores provide a trait-level marker of whether the intervention’s repeated micro-affordances generalized into durable, perceived relational embeddedness. The SSBS has also demonstrated strong internal consistency (*α* = 0.96; Whiting et al., 2017).

Momentary (state-level) regulations will be assessed through a daily, single-item Ecological Momentary Assessment (EMA) self-rating prompt delivered throughout the eight-week intervention phase. Adolescents are prompted with the question: “Right now, how effectively are you managing your emotions and behavior?” and respond on a 1–7 Likert scale (1 = *not effectively at all*, 7 = *very effectively*). Basically, this item is designed to capture real-time regulatory enactment rather than a retrospective or global self-assessment. Thus, EMA state trajectories recorded across Phase B provide a mechanism-level time-series dataset optimally structured for single-case Tau-U analysis.

All outcome indicators depend on student self-report, the protocol also incorporates two additional informant-based measures. Teacher behavioral ratings will be collected at pre- and post-intervention timepoints using a brief four-item adaptation of the Social Skills Improvement System (SSIS; Elliott et al., 2008) targeting observable classroom regulation behaviors (e.g., “This student recovers quickly from frustration”) and prosocial conduct (e.g., “This student initiates positive interactions with peers”). Items are rated on a 4-point frequency scale (1 = never, 4 = almost always) by the student’s primary classroom teacher. Parent or guardian reports will be collected using a parallel four-item brief form targeting home-context regulation and social behavior, administered via a secure online survey link at the same two timepoints. These informant sources provide convergent evidence from ecologically distinct observational contexts, reducing the sole reliance on student self-report and mitigating single-source inflation of perceived effects.

Implementation of fidelity will be captured via weekly counselor fidelity summaries. To minimize administrative burden and avoid interpretive drift, this documentation uses a structured, three-item binary checklist. The counselor simply records whether: (1) micro-nudges were opened at least four times during the week; (2) the student submitted at least one reflective note; and (3) the student concretely described at least one pro-social or regulation-aligned behavior. Counselors may optionally add a single-sentence contextual remark if relevant. These logs do not serve as qualitative data for phenomenology, but rather as direct fidelity evidence confirming that the designed mechanism was actively enacted.

### Intervention content and delivery procedures

This section specifies the full content of the eight-week intervention, integrating the weekly developmental targets, daily MI-style micro-nudges, and dual-trigger micro-coaching dialogues. The primary purpose is to translate the theoretical arc into an operationalizable schedule of high-frequency developmental affordances that can be implemented within standard high school counseling environment while maintaining the strict methodological transparency required for rigorous outcome evaluation. As outlined in Table 1, the weekly architecture includes: (a) a thematic PYD-linked developmental objective; (b) daily AI-delivered micro-nudges (≤ 40 words); and (c) a synchronous 10–15-min counselor mini-session. Sample scripts for the MI-style nudges and the underlying algorithmic logic for the micro-coaching are detailed in the Appendix.

### Research design

The present study utilizes a feasibility-oriented outcome research design rather than a powered efficacy trial. The primary analytic purpose is to demonstrate whether an AI-augmented PYD delivery scaffold can be successfully implemented within standard school counseling environments. Furthermore, it seeks to observe whether the targeted micro–behavioral processes (awareness, micro–choices, regulation moves, prosocial micro-behaviors) the model is designed to generate occur under naturalistic conditions. Grounded in feasibility logic (de Boer et al., 2025), the primary inferential question is not whether the intervention outperforms existing protocols, but rather whether this model can be successfully implemented and whether it reliably produces mechanism-consistent enactments.

A mixed methodological structure is required because the targeted constructs, self-regulation and perceived social connectedness, have a dual ontological status: they exist simultaneously as trait markers (i.e., stable, longitudinal dispositions) and state performances (i.e., enacted, momentarily observable micro-behaviors). Quantitative data alone cannot verify state enactment; rather, process-embedded qualitative evidence is essential to determine whether actual behavioral practice occurred. Consequently, in this protocol, quantitative indices are utilized to capture directional change, while qualitative materials serve to continuously verify mechanism enactment in real time.

Quantitatively, the design combines a within-subject trait-level pre–post comparison with an embedded single-case AB probe. The sequence consists of a pre-intervention baseline (Phase A; Weeks −2 and −1) and an active intervention period (Phase B; Weeks 1–8). The two-week baseline was adopted. A minimum of 10 data points in Phase A provides a more stable trend estimate for Tau-U baseline correction and more clearly distinguishes pre-existing directional drift from intervention-related change. Throughout Phase B, state-level EMA micro-ratings (1–7 Likert scales) index continuously index momentary regulation states. Trait-level self-regulation and social connectedness are measured at pre- and post-intervention timepoints via the within-subject standardized mean difference (Cohen’s *d*). To rigorously capture the dynamics of the state-level micro-trajectories, data will be evaluated using Tau-U, a robust, best-practice non-overlap effect size optimized for AB sequences in single-case experimental design (SCED) methodology.

The qualitative strand consists of (a) weekly counselor fidelity logs and (b) brief, one-sentence reflective student notes. These materials are not narrative interviews; rather, they serve as real-time process-evidence. Therefore, the analytic objective is not to achieve thematic saturation, but to execute mechanism-alignment detection. Reflexive thematic analysis will be used to identify and verify whether developmental practice indicators outlined in Table 1 occurred within a real-world educational environment.

All reflective notes remain accessible to the counselor to preserve the core ethical principle of human relational containment. The AI system does not store raw text beyond the generation of summary logs. Data retention adheres strictly minimal-retention feasibility norms; export functions are disabled. No participant data or conversational data is used for model training or model improvement. Furthermore, no algorithmic action is permitted without the possibility of immediate human override; should emotional dysregulation signals emerge, counselors escalate the case in accordance with established institutional safeguarding protocols. In this design, the AI is never the locus of clinical decision; rather, it is the engine for prompt density. Human counselors retain absolute safeguarding authority and interpretive control.

#### AI system specification

Deployment uses a conversational Large Language Model (LLM) constrained to MI-style reflective prompts. Therefore, the following six aspects of the AI system are specified. (1) System architecture: the system is built on a hosted LLM API (e.g., GPT-4o or equivalent) accessed via a secure HTTPS endpoint. A structured prompt layer constrains all outputs to MI-style reflective responses; the model has no capacity to issue clinical directives. A school-side web application manages student authentication, prompt scheduling, and log aggregation. The counselor receives a read-only dashboard displaying aggregated weekly summaries. (2) Inference mechanisms: at each interaction, the system retrieves the student’s current weekly objective from the protocol schedule and selects a prompt template from the versioned library. No personalized inference or predictive modelling is performed; prompt selection is rule-based and fully deterministic, which eliminates black-box decision risk. (3) Data management: all prompt templates and micro-nudge libraries are hash-versioned to ensure reproducibility. Weekly interaction logs store only aggregated counts and engagement summaries (i.e., whether a prompt was opened and whether a note was submitted); raw conversational text is not retained beyond the session. Counselor fidelity logs are stored separately on the school’s own server and are not transmitted to the AI provider. (4) Privacy protection: the system is designed to comply with applicable data protection frameworks (e.g., GDPR, FERPA). No personally identifiable information is transmitted to the LLM API; each student is represented by a pseudonymous ID. Parental or guardian consent is required for all participants under 18. All data in transit are encrypted; data at rest are stored on school-controlled infrastructure. (5) Update procedures: prompt libraries and system instructions are updated only between academic cohorts, not during an active protocol cycle. Any update triggers a full version increment and requires sign-off by the research team prior to deployment, ensuring within-study consistency. (6) Algorithmic audit: prior to deployment, the full prompt library is reviewed by at least two independent raters for cultural sensitivity, developmental appropriateness, and potential for harmful outputs. Banned-topic filters and automated crisis keyword detection are evaluated against a benchmark set of at-risk phrases; any keyword match triggers an immediate handoff to the human counselor. Post-hoc audit logs are retained for the duration of the study to support independent review.

To ensure flexibility and continuity of engagement, students access the AI micro-nudge system using their own personal devices (e.g., mobile phones, tablets, or home computers). To uphold digital equity, school terminals remain accessible as an alternative for students without personal access. The platform also integrates comprehensive accessibility features including screen-reader compatibility, high-contrast text options, and optional multilingual delivery to support equitable participation.

### Data analysis strategy

The analytical framework of this study is structured to evaluate three outcomes: (a) trait-level directional movement, (b) state-level micro-trajectory change, and (c) mechanism enactment evidence. These three analytical layers correspond directly to the theoretical dimensions the intervention engages in disposition, enactment, and meaning-in-use. To assess trait-level shifts, pre- and post-intervention changes in the BSCS and SSBS will be quantified using a within-subject standardized mean difference, following Lakens’s (2013) generalized within-subject effect size formulation. This approach mitigates dependence on between-subject variability and is appropriate for feasibility designs wherein each participant serves as their own baseline control (Lakens, 2013, 2017). To robustly index the magnitude of directional movement, 95% confidence intervals will be calculated, despite traditional null-hypothesis significance testing not being required under feasibility logic.

State-level EMA sequences (Phase A baseline vs. Phase B intervention) will be analyzed using Tau-U with baseline trend correction. Importantly, correcting for baseline trend prevents inflated effect-size estimates that might otherwise be misattributed to intervention exposure rather than pre-existing directional drift. Tau-U allows non-overlap evaluation even with small samples and is currently the most methodologically defensible effect-size metric for SCED micro-regulation sequences. Qualitative data will be evaluated via reflexive thematic analysis, though explicitly restricted to mechanism verification rather than narrative phenomenology. Coding will be deductive and anchored to the weekly objective architecture.

Finally, to integrate the three evidence streams, a triangulation matrix will be constructed to map (a) trait-level shifts, (b) state-level effect estimates, and (c) qualitative mechanism evidence. The purpose of this synthesis is not to blend subjective narrative with statistical data, but to evaluate whether the targeted mechanism signal is reliably detectable across heterogeneous measurement modes. Methodological convergence across these domains confirms intervention feasibility, while signal divergence indicates a structural implementation malfunction.

#### Missing data handling

Missing data for the trait outcomes (BSCS, SSBS), analyses will be managed via pairwise availability with sensitivity checks using last-observation-carried-forward for descriptive ranges. For the state-levelEMA data, missing days will simply be omitted because Tau-U inherently tolerates unequal phase lengths and missing data points within time-series sequences. To rigorously assess the effect of intervention dosage, per-participant compliance (percentage of EMA prompts answered) will be reported and descriptive contrasts comparing high- compliance (≥ 60%) versus low- compliance (< 60%) will be conducted.

### Practical contribution

The primary practical contribution of this feasibility protocol is not to establish causal efficacy, but in solving a foundational translational problem in implementation science. Complex socioemotional interventions often fail in fully powered randomized controlled trials (RCTs) not because the underlying developmental theory is flawed, but because the delivery mechanisms are incompatible with real-world school environments. By successfully mapping relational developmental systems theory onto an actionable digital delivery system, this protocol provides a rigorously specified protocol and open-source methodological blueprint that future researchers can adopt as an intervention for fully powered RCTs. It forms links between abstract theoretical goals and concrete, ecological implementation. Ultimately, this model functions as a transferable developmental scaffold specifying developmental arc, AI-augmented dosage logic, human-in-the-loop safety structure, and measurement architecture necessary for rigorous clinical replication. By establishing clear operational compliance targets, setting ethical boundary conditions for artificial intelligence, and illustrating how to synthesize quantitative time-series metrics with qualitative mechanism evidence, this protocol significantly reduces the friction for future outcome research. Researchers and clinicians can adapt it across different adolescent populations and diverse socioeconomic and cultural settings, alternative developmental targets, or varied educational environments, thereby accelerating the field’s progression toward rigorous, large-scale efficacy testing of AI-supported and AI-augmented developmental interventions.

### Limitations

Given its architecture as a feasibility design, this study cannot causally attribute observed changes to the intervention, nor does it make any statistical inference regarding comparative superiority or clinical efficacy. Any observed trait- or state-level shifts may be confounded by normative developmental maturation, placebo engagement, social desirability, technological novelty effects, or seasonal artifacts of the school calendar. Furthermore, the fidelity documentation is strictly designed to assess the mere presence of mechanism enactment, not the qualitative depth or mastery of the skill. Consequently, no estimates of generalizability are proposed or assumed.

In addition to the design-level constraints noted above, several AI-specific risks warrant explicit acknowledgment, as these are now priority concerns in educational AI literature. First, LLMs are susceptible to hallucination, meaning the model may occasionally generate factually inaccurate or contextually inappropriate responses. Although the system’s rule-based prompt architecture constrains output to MI-style reflective templates and reduces this risk, it cannot be entirely eliminated. Counselors are therefore instructed to review flagged interactions and to treat any factually dubious AI output as a fidelity concern requiring immediate intervention. Second, algorithmic bias may be present in the underlying model’s training data. Students from minority cultural, linguistic, or socioeconomic backgrounds may encounter prompts that implicitly reflect dominant-cultural norms or fail to resonate within their relational context. The pre-deployment audit described in the AI System Specification section includes sensitivity review by raters with diverse cultural backgrounds; however, residual bias cannot be fully excluded and should be documented during implementation. Third, emotional dependence on the AI system is a theoretically plausible risk, particularly for adolescents experiencing social isolation or acute relational distress. The protocol mitigates this risk structurally by maintaining the weekly counselor mini session as a non-negotiable relational anchor and by ensuring the AI’s role is framed to students as a practice tool rather than a relational agent. Counselors are trained to monitor engagement patterns and to intervene if a student’s interaction frequency suggests substitution of AI contact for peer or counselor relationships. These risks do not invalidate the feasibility rationale of the study, but they underscore the necessity of maintaining robust human oversight throughout the protocol.

### Future directions

Future research should move beyond evaluating whether AI-augmented interventions “work” and begin examining how developmental mechanisms unfold across ecological time. In PYD-oriented intervention science, this shift may allow researchers to conceptualize development not as a static pre–post outcome, but as a dynamic sequence of micro-enactments occurring across everyday contexts. AI-supported EMA systems may therefore contribute to a broader methodological transition from episodic intervention models toward continuous developmental process monitoring.

Within PYD literature specifically, future studies may explore whether AI-enhanced delivery systems can strengthen underexamined developmental pathways such as resilience transfer, identity coherence, future orientation, and culturally contextualized belonging. The flexibility of AI-supported micro-interactions may also enable more adaptive and personalized developmental scaffolding, including culturally responsive framing, language-sensitive prompts, and differential pacing based on student engagement patterns. Such possibilities are particularly relevant for school counseling systems that operate under high relational demand and limited institutional capacity.

Future work should additionally investigate whether AI-assisted interventions can support relational continuity rather than merely increasing digital interaction frequency. Rather than conceptualizing AI as a substitute for counselor expertise, subsequent research may examine how AI can preserve counselor emotional bandwidth by redistributing repetitive micro-support tasks while maintaining human interpretation, ethical judgment, and developmental attunement as central features of care.

At the methodological level, future studies may integrate passive sensing data, adaptive intervention sequencing, and reinforcement-learning informed prompt timing to examine whether developmental support can become increasingly context-responsive without compromising ethical safeguards. However, these advances should be approached cautiously. Future AI-based psychological interventions must continue prioritizing transparency, human oversight, bias mitigation, and youth protection frameworks, particularly in school-based contexts involving minors. Ultimately, the long-term contribution of AI in PYD research may not lie in replacing human developmental relationships, but in expanding the temporal and ecological reach of those relationships in ways that were previously impractical within conventional counseling delivery systems.

## Conclusion

This paper presented a study protocol and outcome research design for an AI-augmented PYD intervention intended for school counseling settings. Rather than positioning artificial intelligence as a replacement for human counseling relationships, the proposed model conceptualizes AI as a structured developmental support mechanism capable of extending the frequency and ecological reach of reflective micro-interactions within adolescents’ daily lives. By integrating PYD theory, mixed-method outcome research, EMA, and SCED methodology, the paper offers a transparent and replication-ready framework for future feasibility and controlled intervention studies. More broadly, the proposed design contributes to emerging discussions regarding how psychological science can responsibly integrate AI into educational and counseling practice without diminishing the importance of human judgment, relational attunement, and ethical oversight. As schools continue to face increasing developmental demands alongside limited counseling capacity, AI-supported intervention architectures may offer one pathway for expanding developmental opportunities while preserving the counselor–student relationship as the central foundation of care.

## Appendix

### Appendix A: sample micro-nudge scripts (week examples)

Week 1 – Awareness: “Notice one emotion you felt today. Name it. That alone is a skill.”

Week 3 – Regulation Moves: “When tension rises, take one slower breath than usual. Small slows count.”

Week 5 – Prosocial Action: “Choose one moment to be intentionally kind today. Micro is enough.”

### Appendix B: counselor fidelity log template

□ Student opened ≥4 nudges this week

□ Student submitted ≥1 reflective note this week

□ Student described ≥1 behavior aligned with weekly objective

Optional one-sentence remark: ____________________________.

### Appendix C: example micro-coaching dialogue (3–5 turns)

AI: “What small moment today challenged you most?”

Student: “During break they ignored me.”

AI: “What choice did you make in that moment?”

Student: “I walked away.”

AI: “Was that choice aligned with how you want to show up socially?”
